# Preparation of mechano-nanoswitches for ultrasound-controlled drug activation

**DOI:** 10.52601/bpr.2024.240054

**Published:** 2025-10-31

**Authors:** Zhihuan Liao, Junliang Chen, Menghan Xiao, Shuaidong Huo

**Affiliations:** 1 Fujian Provincial Key Laboratory of Innovative Drug Target Research, School of Pharmaceutical Sciences, Xiamen University, Xiamen 361102, Fujian, China

**Keywords:** Ultrasound, Mechano-nanoswitch, Nanodimer, Drug activation, Force-sensitive

## Abstract

Chemotherapy is often hindered by issues associated with deficient drug selectivity and ineluctable toxic effects. The emerging realm of mechanochemistry has demonstrated significant promise in precise drug activation by using ultrasound-induced mechanical forces to regulate the chemical properties of compounds at the molecular level. Recently, we proved that the successful introduction of nanostructures to mechanochemistry could improve drug loading capacity and enhance their mechanical responsiveness. To further expand the application of the ultrasound-response drug activation strategy in nanosystems, in this context, we illustrate the preparation of a mechano-nanoswitch for spatiotemporal control of drug activation.

## INTRODUCTION

Controlled drug release is a promising strategy for improving therapeutic efficacy while reducing side effects (Baryakova *et al*. 2023; Mitchell *et al*. 2021; Stater *et al*. 2021). In recent years, drug delivery systems responsive to specific stimuli have been developed to regulate drug release in response to internal triggers or external stimuli (Fan *et al*. 2023; Mi 2020). However, without precise control over drug activity, these approaches face limitations, such as premature drug leakage, low response sensitivity, *etc*. (Tu *et al*. 2021).

The emergence of mechanochemistry brings new possibilities for altering drug activity by utilizing ultrasound-induced shear force to trigger specific chemical bond cleaving or rearranging (O’Neill and Boulatov 2021; Zhao *et al*. 2021). Recently, we presented the first example of ultrasound-induced mechanochemical bond cleavage for drug activation, demonstrating the potential of ultrasound for spatiotemporal control of drug activity (Huo *et al*. 2021). Later, we showed that combining nanoparticle systems with polymer mechanochemistry improves drug loading capacity and significantly enhances mechanical responsiveness (Huo *et al*. 2022). Taking the reported gold nanodimer and anticancer drug doxorubicin (DOX) as an example, herein, we provide a detailed description of the protocol for constructing a mechano-nanoswitch that selectively activates drugs by ultrasound. The nanoparticles at both ends serve as the conductive arms of the sonomechanical force, while the drug loading site in the middle is the mechanophore (force-responsive group). In theory, the relevant parts can also be adjusted or replaced with other nanostructures and mechanophores accordingly. Due to the particularity of the nanodimer coupling and preparation process, the yield of the nanodimer is about 8%. High-purity nanodimers can be obtained through a straightforward gel electrophoresis, greatly simplifying the purification process. This protocol provides an approach for creating mechanosensitive nanosystems, offering more precise control over drug activity and valuable insights for future applications in nanomedicine.

## STEP-BY-STEP PROCEDURE

### Step 1: Preparation of gold nanoparticles (AuNPs) [TIMING 2–3 d]

Step 1.1: Prepare aqua regia in a large beaker in a fume cupboard by mixing 3:1 (*v*:*v*) concentrated HCl:HNO_3_. Soak utensils that come into contact with AuNPs during synthesis in aqua regia for at least 15 min. Rinse the utensils with plenty of deionized water and then with Milli-Q water (Liu and Lu 2006).

**[TIP]** Obtaining high-quality AuNPs is the first step toward experimental success. Take care to ensure that no contamination is introduced during the AuNPs synthesis process.

**[CAUTION]** Be extremely careful when preparing and using aqua regia. Wear goggles and gloves, and conduct the experiments in a fume cupboard. Aqua regia should be prepared freshly and never stored in closed containers.

**Figure 1:**
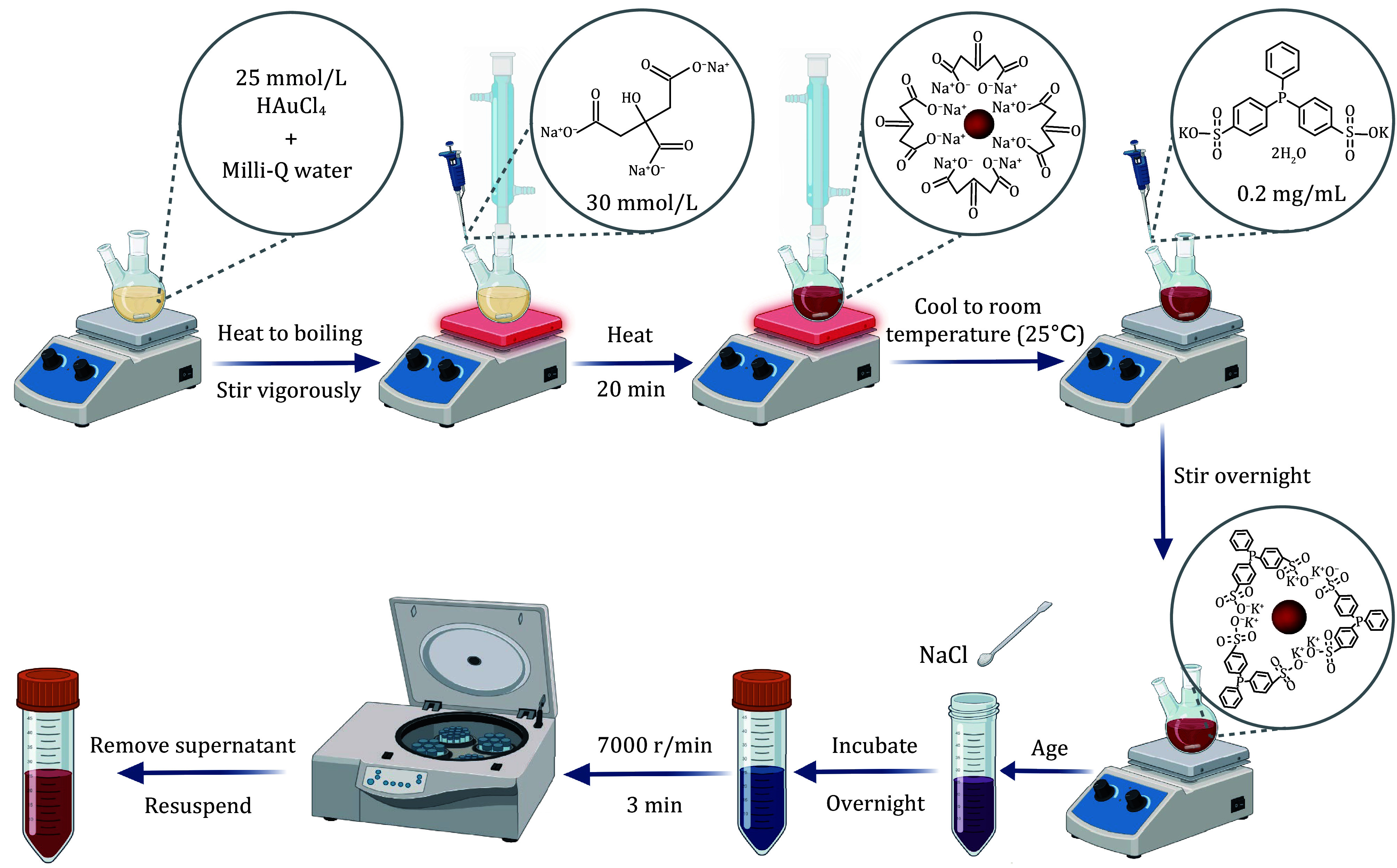
The workflow of preparation of AuNPs

Step 1.2: Load 30 mL of Milli-Q water in a two-necked flask. Add 0.51 mL of 25 mmol/L HAuCl_4_ solution. Place the flask on a hotplate and reflux while stirring vigorously. Wait for the solution to boil, then add 1.8 mL of citric acid solution (30 mmol/L) quickly, and the heating ceases after 20 min, then cool naturally to room temperature (25°C) to form the 15 nm citrate-protected AuNPs (Fig. 1).

**[TIP]** The size of nanoparticles plays an important role in their mechanical response. In general, the larger the particle size, the more pronounced its mechanical response. The particle size selected in this protocol is around 15 nm to provide a typical case commonly used.

Step 1.3: Add Bis(p-sulfonatophenyl)phenylphosphine dihydrate dipotassium salt (BSPP) rapidly to the mixture to reach a concentration of 0.2 mg/mL and stir thoroughly overnight at room temperature (25°C) to facilitate the modification of the AuNPs surface.

**[TIP]** The principal role of BSPP is to facilitate stability and surface charge optimization for AuNPs, thus providing a stable and negatively charged surface for subsequent DNA binding. This step enhances the efficacy and reproducibility of experimental procedures (Jin *et al*. 2015).

Step 1.4: Add solid sodium chloride (NaCl) to the modified AuNPs until a color change from red to blue is observed. The addition of NaCl shields the surface charge of the AuNPs, resulting in AuNPs aggregation (solution color changes from red to blue). Then, incubate the mixture overnight at room temperature (25°C) to ensure complete interaction. Centrifuge the mixture at 7000 r/min for 3 min to remove the supernatant. After removing the salt solution, resuspend with Milli-Q water to complete the aging process of the AuNPs. When NaCl is removed, the surface charge is restored and the particles are dispersed again (solution color returns to red).

**[TIP]** Perform this step directly with a 50 mL centrifuge tube to improve the aging efficiency. At this point, the AuNPs will be collected at the bottom of the centrifuge tube. Carefully remove the supernatant and do not remove the AuNPs.

### Step 2: Preparation of Au-DNA dimer mechano-nanoswitches [TIMING 2–3 d]

**Figure 2:**
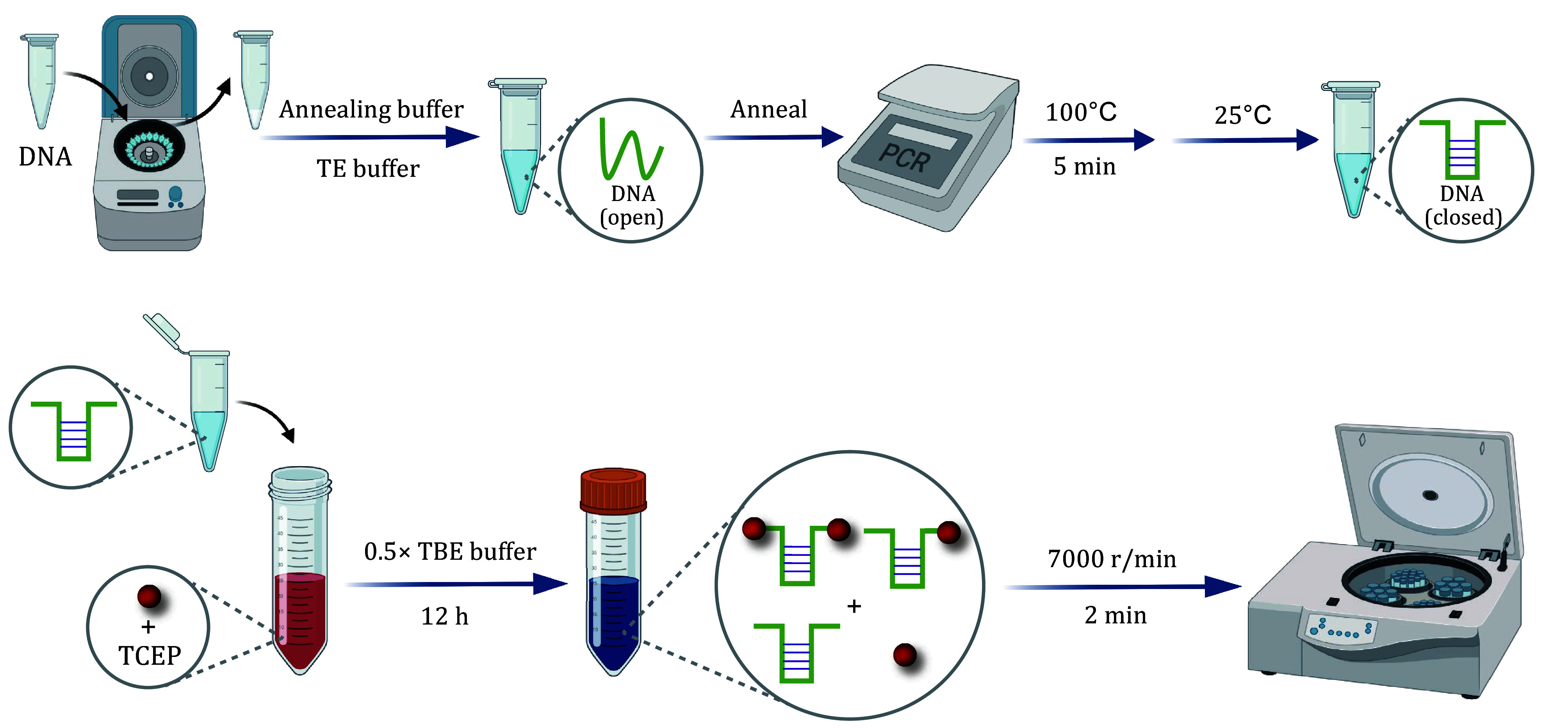
The workflow of preparation of Au-DNA dimer mechano-nanoswitches

Step 2.1: Dissolve the terminally double-thiolated DNA aptamer in annealing buffer (200 mmol/L KCl, 4 mmol/L MgCl_2_, and 28 mmol/L Tris-HCl), and then anneal at 100°C for 5 min. Cool the mixture slowly to 25°C to form a double-stranded DNA structure (closed configuration) (Fig. 2).

**[TIP]** Before dissolving with buffer solutions, centrifuge the test tube containing aptamer powder at 4000 r/min and collect to the bottom of the tube. Once the stocking solution is prepared, it should be stored at 4°C.

Step 2.2: Prepare Au-DNA conjugates by mixing AuNPs with the aptamer (TCEP treated) in a molar ratio of 3:1, and incubate the mixture in 0.5× TBE buffer (containing 50 mmol/L NaCl) for 12 h. Adding TBE buffer may cause AuNPs aggregation, and the solution may turn red to blue. Following incubation, centrifuge the solution at 7000 r/min for 2 min to facilitate the separation of the supernatant, which is then carefully removed. After eliminating the salt solution, concentrate the remaining solution to approximately 20 μL.

**[TIP]** Before coupling with AuNPs, introduce tris(2-carboxyethyl)phosphine hydrochloride (TCEP) to reduce the disulfide bonds present in the primer. This step is crucial to prevent potential cross-linking between the primers’ terminal sulfhydryl groups and the polymer during subsequent annealing operations.

### Step 3: Purification of Au-DNA dimer mechano-nanoswitches [TIMING 2-3 d]

**Figure 3:**
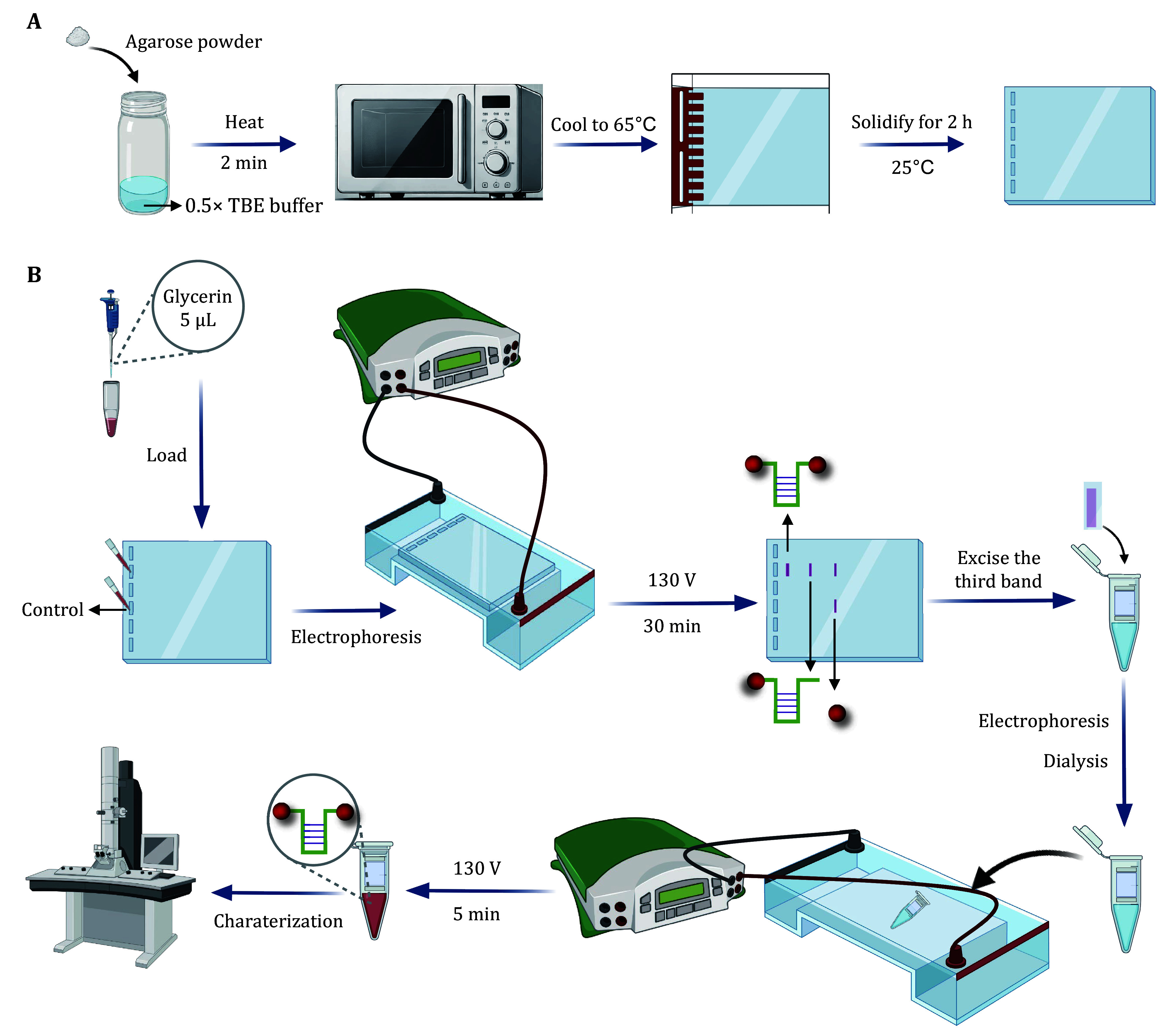
The workflow of agarose gel preparation (**A**) and purification of Au-DNA dimer mechano-nanoswitches (**B**)

Step 3.1: Weigh 1.2 g of agarose powder and mix with 40 mL of 0.5× TBE buffer to achieve a 3% (*w*/*v*) agarose concentration. Shake the mixture thoroughly and then heat in a microwave oven for 2 min until the agarose is completely dissolved. After heating, cool the solution to approximately 65°C. Subsequently, pour the agarose solution meticulously into a casting tray and cool naturally. Insert a comb vertically into the gel to create wells, and solidify the gel at 25°C for 2 h (Fig. 3A).

**[TIP]** Clean the gelatine mold meticulously each time to prevent contamination. It is essential to ensure that the melted agaric is adequately shaken and meticulously poured onto the gelatin plate, as this will minimize the risk of obtaining suboptimal results later. The selection of an appropriate agarose concentration is of critical importance for the successful isolation of Au-DNA dimers. Based on the results of our experimental trials, 3% (*w*/*v*) agarose was identified as the optimal gel concentration.

Step 3.2: Mix 15 μL Au-DNA conjugates solution with 5 μL glycerin and load gently into an agarose gel. Then, carefully load the solution into the wells of the agarose gel. As a control, AuNPs that have undergone the aging process but without modification with aptamer are also included in the experiment. The electrophoresis is conducted at a voltage of 130 V for 30 min in a 0.5× TBE running buffer within the electrophoresis tank (Fig. 3B).

**[TIP]** The electrophoresis tank provides sufficient coverage of the agarose gel, with approximately 20 μL per well. Furthermore, the inclusion of glycerol in the mixture is recommended to prevent the contents of the wells from migrating out during the electrophoresis process. This precaution is to maintain the samples’ integrity and achieve clear and distinct target bands for analysis.

Step 3.3: Following electrophoresis, meticulously remove the agarose gel and carefully cut the third target band. Subsequently, recover the cut band with a D-Tube™ electroelution accessory kit through another gel electrophoresis at 130 V for 5 min. Place the kit horizontally in the electrophoresis bath, and add Milli-Q water until the gel bands are fully submerged.

**[TIP]** The electrophoresis time for the electroelution accessory kit is not excessive to prevent the gel from dissolving, which could have a detrimental impact on subsequent characterization.

### Step 4: Doxorubicin intercalation into mechano-nanoswitches [TIMING 1 d]

**Figure 4:**
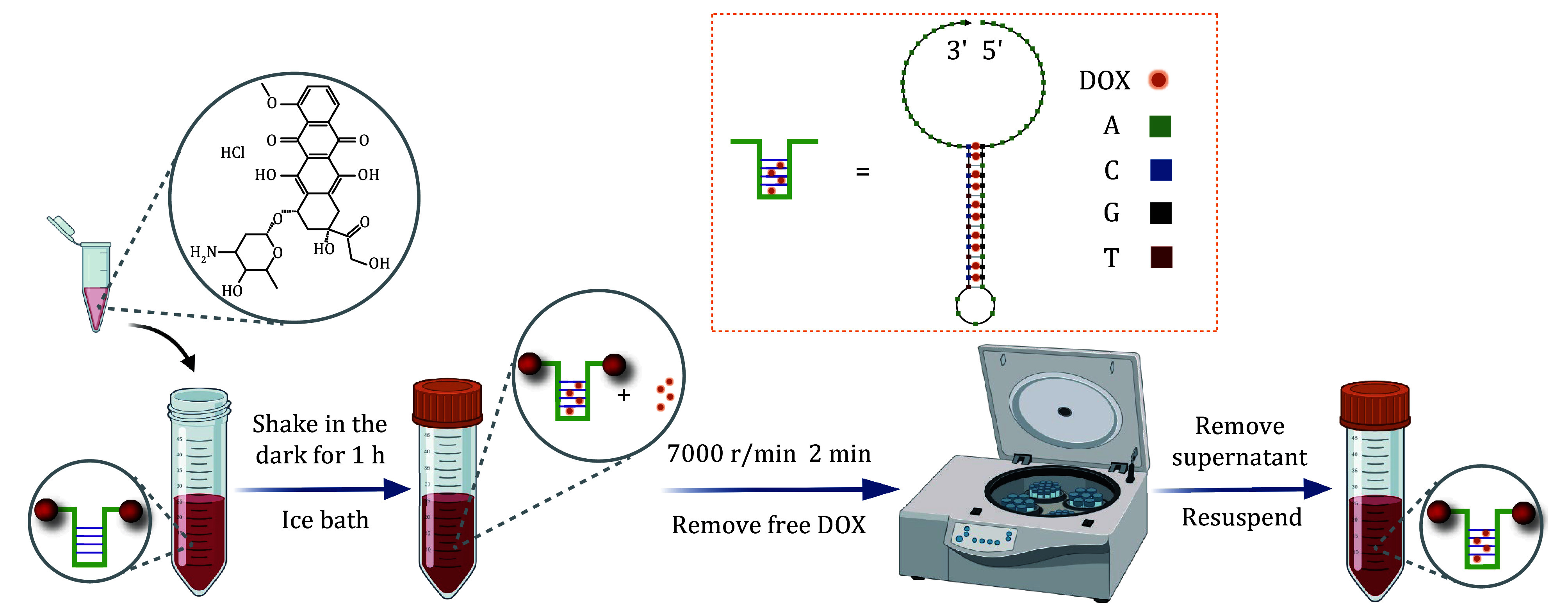
The workflow of doxorubicin intercalation into mechano-nanoswitches

Step 4.1: For the doxorubicin (DOX) intercalation, incubate DOX with the Au-DNA dimer at a molar ratio of 20:1 and shake gently in the dark for 1 h in an ice bath. The DOX is capable of intercalating into double-stranded 5’-GC-3’or 5’-CG-3’ (Zhu *et al*. 2013). After that, centrifuge the complex to remove excess free DOX (Fig. 4).

The DOX loading efficiency is calculated based on the following formula:

DOX loading efficiency (%) = [1 − (Fluo_free_ − Fluo_buffer_)/　　　　　　　　　　　　 (Fluo_total_ − Fluo_buffer_)] × 100%

**[TIP]** The incubation ratio and time can be adjusted accordingly with the number of drug intercalation sites in the designed DNA sequence.

### Step 5: Ultrasound-controlled drug activation experiments [TIMING 1-2 d]

**Figure 5:**
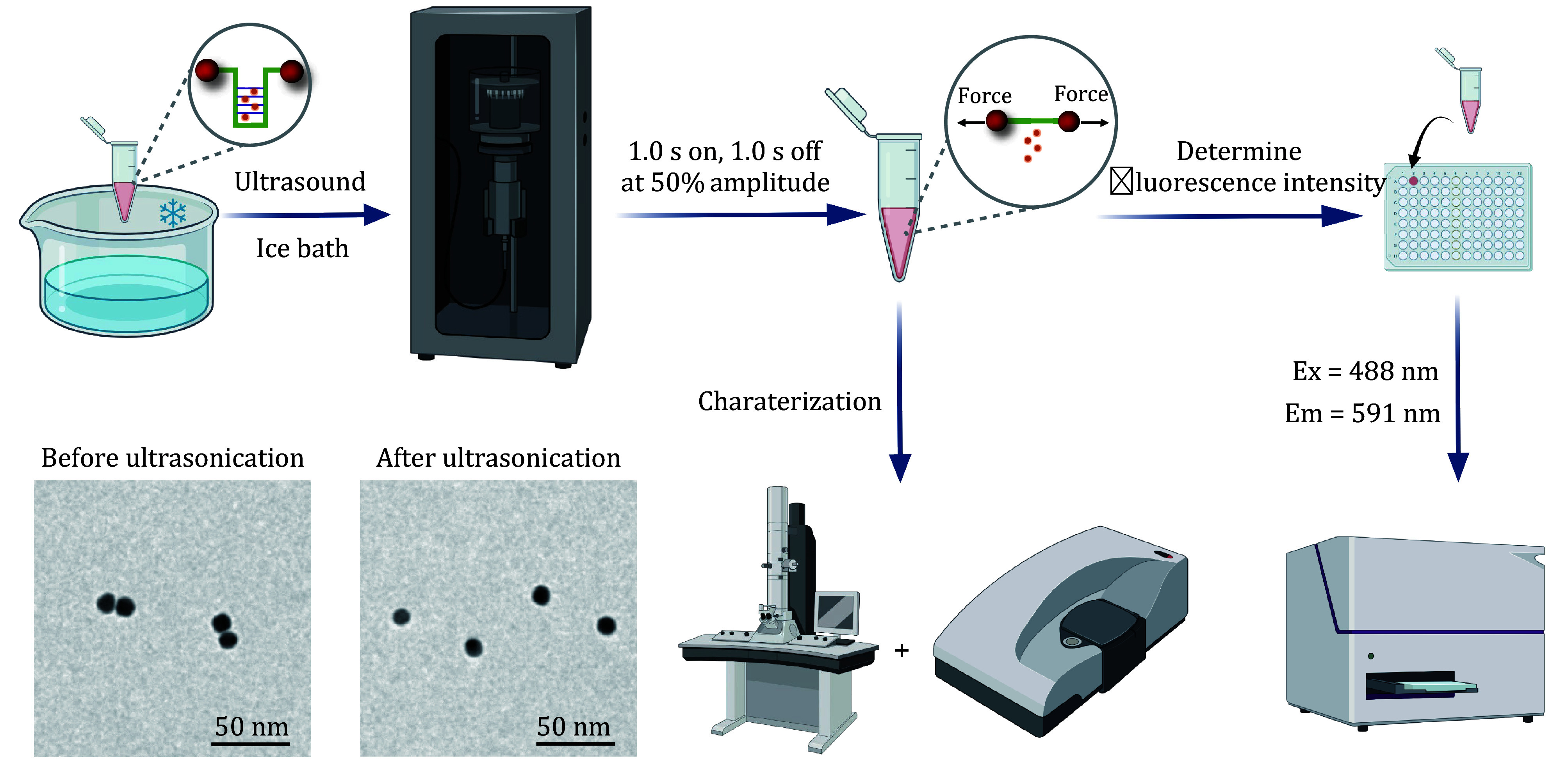
The workflow of ultrasound-controlled drug activation experiments

Step 5.1: Perform an ultrasonication experiment of the DOX-loaded mechano-nanoswitches in a 1 mL heavy-walled ultrasonication vessel with a sonicator equipped with a 3 mm diameter microtip probe (A12628PRB20). Perform sonication using pulsed ultrasound (1.0 s on, 1.0 s off at 50% amplitude) at *f* = 20 kHz. Place the vessel in an ice bath to maintain a temperature inside the vessel of 6–9°C throughout sonication (Fig. 5).

**[TIP]** To prevent the thermal effects of ultrasound from influencing the structure of mechano-nanoswitches, the sample should be kept in an ice bath throughout the sonication experiment.

Step 5.2: For studying the mechanochemical response of the drug (DOX) release, measure the fluorescence emission intensity of the supernatant at *λ* = 591 nm immediately after ultrasonication, under an excitation wavelength *λ* = 488 nm at 25°C.

Step 5.3: For studying the US-induced structure change of the mechano-switches, deposit 4 μL of the post-dialysis solution onto a copper mesh, evaporate, and dry naturally. Then observe the sample under a transmission electron microscope (TEM). Meanwhile, determine the particle size change through dynamic light scattering (DLS) analysis.

**[TIP]** Conduct cell toxicity or animal experiments to verify the activity of the released drugs. Select other characterization methods to analyze the regulatory behavior of sonomechanical force on drug activity based on the type and characteristics of the drugs loaded.

## MATERIALS AND EQUIPMENT

### Materials

• Chloroauric acid (Sigma) CAS No. 27988-77-8

• Sodium citrate (Sigma) CAS No. 6132-04-3

• Bis(p-sulfonatophenyl)phenylphosphine dihydrate dipotassium salt (Sigma) CAS No. 308103-66-4

• Sodium chloride (Sigma) CAS No. 7647-14-5

• Agarose (Sangon Biotech) CAS No. 9012-36-6

• DNA (SH-5’-AAAAAAAAAAAAAAAAAAAAGGAGGAGGAGGAGGAAAAATCCTCCTCCTCCTCCAAAAAAAAAAAAAAAAAAAA-3’-SH)

• Doxorubicin hydrochloride (Sigma) CAS No. 25316-40-9

• TBE buffer (Sangon Biotech), 5×

• Tris(2-carboxyethyl)phosphine hydrochloride (Sangon Biotech) CAS No. 51805-45-9

• Ultrasonication vessel (Test tube heavy-walled, 2775/2, Assistant)

• D-Tube™ electroelution accessory kit (D-Tube™ Dialyzer Midi, Merk)

### Equipment

• Magnetic heating agitator (IKA)

• Centrifugal machine (HC-3016R)

• Horizontal electrophoresis apparatus (BIO-RAD)

• Transmission electron microscopy (JEOL JEM-2100plus)

• Nano ZS Zetasizer (25°C, Malvern, England)

• Gel imager system (Tanon)

• Qsonica Q125 sonicator (USA)

## Conflict of interest

Zhihuan Liao, Junliang Chen, Menghan Xiao and Shuaidong Huo declare that they have no conflict of interest.
